# The life stories and experiences of the children admitted to the Institute for Imbecile Children from 1895 to 1913

**DOI:** 10.4102/ajod.v9i0.669

**Published:** 2020-08-28

**Authors:** Rory du Plessis

**Affiliations:** 1 School of Visual Arts, Faculty of Humanities, University of Pretoria, Pretoria, South Africa

**Keywords:** Dr Thomas Duncan Greenlees, Makhanda, children with intellectual disabilities, Cape Colony, disability studies, personhood, humanness

## Abstract

**Background:**

South African scholarship on intellectual disability has produced a sizeable body of research, yet there are numerous areas where there is a paucity of research. One area in which there is a conspicuous paucity of research is historical studies of people with intellectual disability (PWID). The existing works devoted to the history of PWID in South Africa are primarily focused on the legal provisions and institutions for the protection and care of PWID. Missing from these works are the life stories and experiences of PWID.

**Objectives:**

The article offers a study devoted to the life stories and experiences of the children with intellectual disability (CWID) who were admitted to the Institute for Imbecile Children from 1895 to 1913. The institute opened in April 1895 in Makhanda (formerly known as Grahamstown), South Africa. The institute was the first of its kind in the Cape Colony for CWID.

**Method:**

The study presents a qualitative investigation of the life stories and experiences of the children that were recorded in the institute’s casebook. The entire set of 101 cases contained in the casebook was analysed by adopting a Gadamerian approach to hermeneutics.

**Results:**

The examination of the institute’s casebook identified several broad themes relating to the children’s admittance, daily life at the institute and their routes out of the institute. The study also extols the individuality of each child’s life story to provide an awareness and richer appreciation of the humanness and personhood of the children.

**Conclusion:**

The article contributes a positive narrative to the identity and the history of South African children with intellectual disability living in the late 19th and early 20th centuries.

## Introduction

More than two decades ago, Anne Digby, a leading social historian of medicine, stated that: ‘[*h*]istorically, the social marginality of people with learning disabilities has been mirrored by their academic marginality’ (Digby 1996:1). Although South African scholarship on intellectual disability has produced a sizeable body of research, one can argue that Digby’s statement is still true as there are numerous areas where there is a paucity of research (see Capri 2016). One area in which there is a conspicuous paucity of research is historical studies of people with intellectual disability (PWID) in South Africa. As far as I can ascertain, works devoted to the history of PWID in South Africa are covered only by Foster (1990) and Minde (1975a, 1975b).

Although these authors’ studies are laudable for doing pioneering groundwork, their primary focus is on the history of the legal provisions and institutions for the protection and care of PWID. Missing from the works of both of these authors are the life histories and experiences of PWID. For Atkinson (2005:10), such an omission serves to endorse a misguided perception that ‘people with learning disabilities [are] seen as a people with “no history”’.

Studies on the history of South Africa’s psychiatric hospitals have offered a glimpse into the life stories of the PWID who were institutionalised patients (e.g. see Swartz 1996). These studies remain praiseworthy for highlighting the life stories of PWID, but they are only a small part of a larger body of research focused on researching the lives of patients suffering from mental illness. In this article, I aim to offer a study devoted to the life stories and experiences of the children with intellectual disability (CWID) who were admitted to the Institute for Imbecile Children from 1895 to 1913.

The Institute for Imbecile Children opened in April 1895 on the grounds of the Grahamstown Lunatic Asylum (hereafter ‘the asylum’) in Makhanda (formerly known as Grahamstown), South Africa. The institute was the first of its kind in the Cape Colony for CWID, and it aimed to ‘educate them, and train them to useful occupations’ (G27–1895:63). Dr Thomas Duncan Greenlees (1858–1929) founded the institute and was appointed as its visiting medical officer from its inception to 1907. In addition to this appointment, Greenlees held the post of medical superintendent of the asylum, from 1890 to 1907, and he was appointed as the surgeon-superintendent of the Chronic Sick Hospital from 1890 to March 1903 (Du Plessis 2017).

A substantial body of documents related to the institute is preserved by the Western Cape Archives and Record Service. The documents include the institute’s annual reports, which were submitted to the government of the Cape Colony, correspondence between Greenlees and government officials, and the institute’s casebook.

Several scientific articles authored by Greenlees (1894a, 1897, 1899, 1903, 1905, 1907) on the subject of PWID complement this body of documents. On closer analysis, the majority of these archived documents and scientific articles can serve little in the way of exploring the life stories of the CWID who lived in the late 19th and early 20th centuries. Although the annual reports and the published scientific articles are invaluable resources in contextualising the conception of the institute, descriptions of the therapeutic regimen offered and the demographic profile of the patients, they are largely silent on the lives of the CWID who were institutionalised. One conclusion that can be made from these documents is that they present a dehumanised construction of the CWID.

In the annual reports for the institute, there is a fixed perception of the CWID as having deficient physical and mental abilities and life stories characterised by neglect and abandonment, where they are deplored for being ‘hopeless and helpless’ (G25–1900:20) cases. To illustrate, Greenlees repeatedly described the children as ‘neglected waifs’ (G27–1896:39), thereby presenting the children as a group who were rejected, ostracised and despised by their families. Greenlees presented a dehumanised construction of the children in the annual reports, but he did concede that in providing a ‘comfortable home and good food’ (G20–1897:25) for the CWID, the institute was ‘doing a good work’ (G20–1897:25). The annual reports of the institute thus indicate that Greenlees realised the need for the facility to be committed to the care and well-being of the children who were admitted. This act of humanitarianism certainly informed the therapeutic regimen of the institute, as the children engaged in amusements and activities and received food, healthcare and material provisions. Nevertheless, the function of the institute in humanitarian terms was only acknowledged in the annual reports and in the asylum’s periodical, *The Fort England Mirror*.^1^

In Greenlees’ scientific articles, he solely envisaged the function of the institute as providing the CWID with ‘the best known methods of educating and training’, with the object of ‘making them useful members of society’ (Greenlees 1897:14). This function was conceived not on humanitarian grounds, but as a means to ensure that a parent’s ‘helpless progeny’ would not become a ‘useless member of society and a burden on the family or the State’ (Greenlees 1907:20). Along these lines, Greenlees held that PWID were objects of contempt and disgust, for burdening their families with the responsibility of caring for them, or for depleting state funds by being admitted to asylums, where they only ‘swell the death rate’ and increase the ‘chronic residuum’, which makes up the patient composition of an asylum (Greenlees 1905:222).

People with intellectual disability were dehumanised not only through their being constructed as undeserving and despised objects of family and state support, but also through the perception that they posed a threat to the ‘health of society as a whole’ (Clarke 2004/2005:74). Greenlees believed that PWID indicated the ‘degeneracy of the Anglo-Saxon race’ (Greenlees 1907:20), and he engaged in divesting them of humanness, by declaring that they were ‘monstrosities’ (Greenlees 1907:21) and an ‘awful curse’ (Greenlees 1899:36). Ultimately, as an advocate of eugenics (Hodes 2015:12),^2^ Greenlees proposed ‘the destruction of infants known to be hereditarily tainted with disease’ (Greenlees 1903:19), as well as ‘sterilisation […] and even the lethal chamber’ (Greenlees 1907:21) for PWID.^3^

To summarise, the institute’s annual reports and Greenlees’ scientific articles are silent on the life histories of the CWID who were admitted to the institute. It is imperative to underscore that the omission or silencing of the children’s life stories is understood as constituting an act of dehumanisation (Gillman, Swain & Heyman 1997:690):

[*I*]t has been argued that people who lack a history are at risk of having a history, and an identity, imposed upon them […]. The objectification of people with learning disabilities as ‘other’ is due in part to this lack of history […]. Seen as an homogenous group, they are defined by their learning disability. (Atkinson 2005:10–11)

One archived resource that offers a means to appreciate and explore the life stories and personhood of the children is the institute’s casebook. Despite the fact that clinical casebooks of PWID are ‘problem saturated and pathologising’ (Gillman et al. 1997:682) and that they predominantly privilege ‘information that is useful to professionals, such as IQ and medical diagnosis’ (Gillman et al. 1997:675), they also contain information about individuals’ unique life stories and distinct personal experiences, and their individuality (Clarke 2006:470; Hoole 2012). Thus, by examining the casebook, we bring into view the humanness of the children.

## Research methods

### Design and data collection

The study presents a qualitative investigation of the life stories and experiences of the CWID that are recorded in the casebook of the Institute for Imbecile Children. The casebook is archived at the Western Cape Archives and Record Service.

### Study population

The institute’s casebook contains the cases of 101 CWID, of whom 51 were female and 50 were male. The average age of the children on admittance was 10 years. The institute was reserved for white children, but in 1908 and 1909 two mixed-race children were admitted. The casebook also records that of the CWID who were admitted, 43 were suffering from epilepsy, 30 were suffering from paralysis and 43 were suffering from mutism. In particular, 10 of the CWID were recorded as being multiply disabled, as they were suffering from epilepsy, paralysis and mutism.

In the institute’s annual reports and casebooks, the children were diagnosed as suffering from either idiocy or imbecility. For historians, idiocy and imbecility are ‘slippery terms’ as they describe ‘a range of behaviours, mental conditions and physical afflictions. Broadly speaking, both conditions were identified in relation to reduced intellectual and functional abilities’ (Eastoe 2019:75). Thus, the diagnosis of idiocy and imbecility lacks a precise graduation of intellectual disability. A further awareness of the slipperiness of the terms becomes apparent when the annual reports and casebook of the institute are scrutinised. For the children who were suffering from mutism and/or epilepsy, the documents of the institute do not clarify whether this was a feature of their intellectual disability or the result of a hereditary or congenital disorder. To substantiate, the annual reports simply stated that ‘physically as well as intellectually … some defect’ (G60–1903:121) was ascertained in a number of children. In the casebook, the pro forma columns for recording whether a child suffered from mutism and/or paralysis required only a yes or no answer.

Although the article does not seek to offer a retrospective diagnosis of the children, where a child is described in the casebook to have ‘significant cognitive difficulties, with little or no apparent understanding of verbal language, little or no ability to care for oneself, and usually associated medical conditions’ (Vehmas 2019:521), I postulate that the child had significant intellectual disability. Furthermore, I also recognise that some of the children were multiply disabled. The study seeks to make special reference to the children who were multiply disabled and/or those with significant intellectual disability, as the vast majority of scholarly enquiries have consigned these children to the ‘margins of disability history’ (Allen & Fuller 2016).

### Data analysis

The entire set of 101 cases contained in the institute’s casebook was analysed by adopting a Gadamerian approach to hermeneutics (see Gadamer 2004). The analysis followed the step-by-step approach for Gadamerian hermeneutics outlined by Fleming, Gaidys and Robb (2003). To explain briefly, this entailed investigating every sentence of the casebook to identify themes and patterns. Once the themes and patterns were identified, the casebook was repeatedly read to develop a detailed contextualisation and comprehension of the themes and patterns pertaining to the children’s life stories and experiences. For Fleming et al. (2003:119), researchers using Gadamerian hermeneutics must ensure that they are ‘responsible for establishing the trustworthiness of the research process and the truthfulness of his or her analysis’, the standards of which pertain to providing the reader with ‘sufficient detail of the processes, as well as the findings in the research report’ (Fleming et al. 2003:119). To this end, the article makes use of extensive quotes from the source material, as well as ensuring that the quotes are sufficiently and coherently contextualised, so that the reader can gain a perspective of the analysed casebook and can thereby establish the credibility of my interpretation.

The Gadamerian analysis embarked upon in this article is delimited to an exploration of the content of the institute’s casebook that pertains to the life stories and experiences of the patients, with the aim of bringing into view their humanity. As such, the article does not claim to offer a complete assessment of the casebook medium, but only reports on the casebook content that presents a ‘snapshot of past lives lived, traces of voice and echoes of experience’ (Eastoe 2020). The clinical content of the casebook and the medical history of the patients are critical sites for future investigation and hold the potential to reveal further levels of interpretative insight and offer a more comprehensive understanding of the casebook medium. In this regard, the analysis and the findings of the article will always be open to alternative and competing interpretations that arise from further scholarship engaging with the clinical content of the institute’s casebook.

### Ethical consideration

The study did not require ethical clearance, as the institute’s casebook is open for public consultation at the Western Cape Archives and Record Service. Where possible, the article uses the current terminology of ‘intellectual disability’, instead of the 19th-century term ‘imbecile’. The article maintains the anonymity of the CWID who were admitted to the institute by using pseudonyms. However, in an effort to humanise the subjects, the children are provided with full names.

## Discussion

The discussion is divided into three sections. The first section considers the admission routes of the children to the institute. The second section discusses the children’s daily life and experiences at the institute. The third section considers the various routes out of the institute.

### Admission routes to the institute

#### Hospitals and welfare organisations

The institute received CWID who were transferred from the hospitals and welfare facilities of the Cape Colony. These can be broadly categorised into two groups, namely, those who were transferred on Greenlees’ request and justification, and those who were transferred to the institute by the authority of the Under Colonial Secretary.

In the first group, admissions came from the Chronic Sick Hospital,^4^ the asylum and St Peter’s Home, a welfare home and orphanage in Grahamstown. Amy Dunn (HGM 24:5) was admitted to the institute at the age of 9 in June 1895 from St Peter’s Home. When she was about 4 years of age, she was found in the town of Addo wandering alone. After several failed attempts to report her whereabouts and identify her parents, she was sent to St Peter’s Home (05 June 1895, CO 7163). Victoria Anderson (HGM 24:10) was originally at a hospital in Kimberley before moving to the care of St Peter’s Home. In 1891, Victoria was sent to the Chronic Sick Hospital, and in April 1895, she was admitted to the institute. Both Amy and Victoria were regarded as patients for whom the education provided by the institute might ‘produce some permanent mental benefit’ (05 June 1895, CO 7163). The siblings Suzanne (HGM 24:45) and Tiaan Bezuidenhout (HGM 24:46) were patients of the Chronic Sick Hospital for several years before being admitted to the institute in October 1901. Greenlees justified their admission to the institute on the basis that it was ‘probable that by education and training they may improve intellectually’ (HGM 24:45). Significantly, in contrast to these cases, where admission to the institute was justified by the prospect of improvement, is the case of Tanya Klopper (HGM 24:29). The 13-year-old Tanya (HGM 24:29) was a patient of the asylum, and owing to her young age, was deemed by Greenlees to be ‘much more suitable for treatment at the Institute […] although there is little hope of improvement mentally’ (23 August 1898, CO 7170).

In the second group, CWID who were patients of the colony’s hospitals were transferred to the institute by the authority of the Under Colonial Secretary from requests made by resident magistrates and the superintendents of general hospitals. Emma Rogers (HGM 24:8) was certified as a ‘lunatic’ and was admitted in 1893 to the Old Somerset Hospital in Cape Town. On 28 November 1895, Emma was admitted to the institute in a poor physical condition, blind and unable to speak. Christine Thompson (HGM 24:14) was a patient of the Old Somerset Hospital from 28 September 1895, before she was promptly sent to the institute on 28 November 1895. The medical certificates for Christine report that she could not speak, she took no notice of her surroundings and she had ‘no conversational or reasoning powers’. Danica Rose (HGM 24:27) was admitted on 01 April 1898 from a Kimberley hospital, where she was receiving treatment in the hospital for some time as a ‘lunatic’. Danica was 16 years old when she was admitted to the institute, and she was suffering from epilepsy and paralysis and was unable to speak.

In the casebook entries for this second group, Greenlees openly criticised the views and practices of the resident magistrates and the hospital superintendents. The cases of Emma Rogers (HGM 24:8) and Danica Rose (HGM 24:27) indicate that these children were certified and were managed as ‘lunatics’. However, Greenlees emphatically declared that CWID ‘are *not* lunatics’ (11 June 1895, CO 7163) (emphasis in original), and he acknowledged the need for resident magistrates to be made aware that ‘[*a*]sylums are not the proper places to treat’ CWID, and for them to become aware of the existence of the institute (HGM 24:27). It was equally imperative for the magistrates to become familiar with the envisaged patient composition of the institute, namely, CWID who were educable and trainable. This would thus exclude the multiply disabled CWID who had been sent to the institute (G60–1903:121).

#### Domestic care and the committal context of children with intellectual disability

The first aim of this section is to explore the domestic care of the CWID evidenced in the casebook. Put differently, prior to institutionalisation, the family was the ‘primary locus of care’ (Rose 2017:16) for CWID, and the presence or absence of this care is documented in the casebook. The second aim is to identify the committal context for the CWID. Although the casebook does not contain the reasons why a family decided to commit their child to the institute, it is possible to identify broad themes that may have influenced their decision.

The neglect and abuse of CWID is evidenced in several cases. Ruan Odendaal (HGM 24:7) was found by the colonial authorities to be living in a ‘dirty and neglected condition’ in an unfurnished house, where he was locked up and left without any supervision. The authorities ascertained that the father was an alcoholic and was widowed, and that when he left for work, he would leave Ruan unattended in the locked-up house. Less severe instances of neglect are evidenced in cases where CWID arrived at the institute in a filthy condition. By way of example, the trio of brothers Isak (HGM 24:31), Janco (HGM 24:32) and Ockert Uys (HGM 24:33) were admitted to the institute on 19 November 1898 in a ‘dirty and neglected condition’. The lack of care is also indexed by guardians who ‘utterly neglected’ the education and learning of their CWID (HGM 24:60).

In a handful of cases, there are instances that can be interpreted as suggesting physical abuse (see also Hoole 2012:223). For example, Hennie Steyn (HGM 24:19) seemed to be continually afraid; he would often run away from adults, and when spoken to, he would raise his hand and arm ‘as if protecting his head from a blow’.

The focus now shifts to the casebook entries that contain multiple indicators of families providing care, being devoted to the well-being of their children and expressing love (see also Clarke 2004/2005:65; Taylor 2017b:763). One recurrent theme in the casebook is CWID who were admitted to the institute in their late teens, where their life stories showed a concerted effort on the part of their parents to ensure that they were, at least to some extent, ‘part of the human community’ (Bogdan & Taylor 1989:145). The casebooks for Susan Pringle (HGM 24:28) and Ethan Johnson (HGM 24:79), admitted to the institute at the ages of 16 and 13, respectively, indicate that both children had attended school for a limited time, even though they ‘did not progress like [the] other children’ (HGM 24:28). Tom Lawton (HGM 24:63) attended school until he was 12 years old, despite suffering from a visual impairment that became severe by the time he turned 14. He was 17 years old when he was admitted to the institute. Before Peter Martins (HGM 24:69) was admitted to the institute, at the age of 14, he had attended school for a while, and when he could not progress anymore at school, his father offered him work. Luke Williams (HGM 24:52) had attended school until he could no longer keep up with his peers. Thereafter, he was kept at home, where he assisted in household duties. He was 18 years old when he entered the institute.

For the parents who were paying for their children’s care at the institute, the casebook contains five cases that indicate how families sought a range of different treatment methods for their children before they admitted them to the institute. Chupik and Wright (2006:83) underscore that such cases serve as an important reminder that ‘most caring (and controlling) of the mentally deficient was occurring outside of formal institutions’, and we hence need to be aware that (Chupik & Wright 2006):

[*T*]he route to the asylum, therefore, was a path that a minority of families did not arrive at without first seeking other types of commonly based medical interventions. Depending upon economic circumstances, location and severity of disability, families relied on a variety of treatments to alleviate their children’s ‘mental deficiency’. (p. 84)

The parents of Sabrina Stone (HGM 24:49) provided her with ‘medical treatment at home’, and only once it was concluded that it ‘gave no good results’ did they seek to admit her to the institute. The medical certificates for Gregory Bowie (HGM 24:50) present a bleak portrayal of him as ‘unable to walk, […] nearly totally blind [and] unable to feed or dress himself’. Yet, his mother was anything but despondent, and she consulted with Greenlees in 1899, 3 years before he was finally admitted to the institute, in June 1902. Even though Greenlees at this first meeting recommended that Gregory be admitted, his mother rejected the offer and tried alternative treatments. Although these treatments ultimately failed, Greenlees acknowledged that his mother did the ‘best she can for him’. In the case of both Sabrina Stone (HGM 24:49) and Gregory Bowie (HGM 24:50), the exact medical treatments are unrecorded, but in the case of Jacques Swanepoel (HGM 24:26) we are provided with the details of one treatment option. Jacques’s parents were citizens of the Zuid-Afrikaansche Republiek, and they resided in Pietersburg (now known as Polokwane). They sought to bathe Jacques in the hot springs of the area as a means of improving his condition. The bathing resulted in some improvement, after which they sought further progress by sending him to the institute.

These cases relate to alternative therapeutic options and home-based medical care, but the following two cases highlight disturbing surgical procedures performed by doctors at general hospitals. Prior to Laura Harvett’s (HGM 24:53) admittance to the institute, her casebook records that she was subjected to ‘two operations performed on head (temporal region) […] to “allow brain to expand”’. In November 1908, Geoff Hunter (HGM 24:82) was admitted to the institute at the age of 10 years, suffering from epilepsy and paralysis. Sometime before his admittance, the casebook documents that he was trephined. Trephining was a surgical procedure that was held by the contemporary medical community to have some credence in the treatment of epilepsy. In particular, Greenlees subjected a patient of the asylum to the surgery as a means to ‘alleviate a cerebral disease giving rise to mental aberration’ (Greenlees 1894b:404).

Section 77 of the *Lunacy Act, 1897*, related to the ‘General Regulations for Institutions for the Care, Education and Training of Idiots or Imbeciles’ (see CO 7559), and it outlined the forms that were required to accompany the admission of a child to the institute. Two of these forms were the ‘Resident Magistrate’s Statement in support of application for admission of a patient’ and the ‘Application of Parent or Guardian for admission of a patient’. Although these forms were not archived, in completing the institute’s casebook, Greenlees provided a summary of their content. Although we must be cognisant that Greenlees’ summary only highlights the content that he deemed important, it still allows us to have a tentative understanding of the committal context of the CWID.

The onset of violent and destructive behaviour is a common theme in the committal context of some of the CWID. Rudi Burger (HGM 24:71), a 12-year-old boy, was admitted to the institute after his behaviour became violent. With Kyle Dawson (HGM 24:89), an 8-year-old boy, his behaviour was not only violent and troublesome, but also led to him ‘frequently [getting] into danger’. With two CWID who were almost 10 years old, their behaviour posed a danger and a threat to other children. Nicole Bredenkamp’s (HGM 24:22) admittance to the institute was preceded by a spate of acts of rage, including setting fire to a house. Julian Cowie (HGM 24:104) was a source of danger to his younger siblings, as he would often strike them with ‘anything he has in his hand’. In all of these cases, the CWID’s age of admission to the institute suggests that the families were providing them with home-based care, but that this ceased when the behaviour of the children became troublesome and posed a danger to themselves and/or others. To illustrate this, Christiaan du Toit (HGM 24:57), who was ‘almost blind’ and ‘deaf mute’, remained in the care of his family until he became ‘[*e*]xcitable and wilful’, after which he was admitted to the institute at the age of 12 years.

Wandering away from home and the risk of getting lost is a second theme in the committal context of the CWID. The 9-year-old Marissa Marais (HGM 24:25) would often lose her sense of direction and wander into the fields by herself. Frik Venter (HGM 24:83), a 14-year-old boy from Knysna who suffered from mutism, would frequently wander away from home, and he was unable to return home unaided. In two cases, the children’s wandering habits were associated with unfavourable domestic circumstances. Shannon Fairhurst’s (HGM 24:30) mother had been dead for some years, and her father was ‘unable to attend to her personally’ and could not curb her wandering habits, as he worked during the day. The father of the siblings Hendrik (HGM 24:43) and Margot Vermaak (HGM 24:44) sought to admit them to the institute, as he had ‘no food for his children’, and ‘one of them had been found wandering away from the home half-clad’ (21 August 1901, CO 7559). Overall, these cases may point to families seeking admission of their children to the institute as a ‘last resort to provide safety and security’ (Taylor 2017a:43). In addition, for families that were struggling from unfavourable circumstances and that were poverty-stricken, institutionalisation of the CWID may have offered a guarantee to the families that their children would have access to food, provision, supervision and healthcare (see also Eastoe 2020).

A third theme is the severity and the frequency of epileptic fits suffered by the CWID. Prior to their admittance, Morgan Jackson (HGM 24:85), age 11, Miencke Jacobs (HGM 24:3), age 12, and Mary Robinson (HGM 24:98), age 7, were suffering from epilepsy, with frequent, and sometimes even daily, epileptic fits. With Amber Mitchley (HGM 24:70), her epileptic fits were so severe that she nearly died on several occasions. The last theme is the predominance of multiply disabled children being admitted at a very young age (see HGM 24:92; HGM 24:23; HGM 24:24; HGM 24:61). These CWID, who were suffering from epilepsy, paralysis and mutism, were all admitted to the institute before they were 10 years old. On the one hand, we can interpret the committal of the young children as being indicative of their families seeking to relieve themselves of caring for the children. On the other hand, it is reasonable to suggest that for the families of multiply disabled CWID, the medical care needs of the children could be provided for better by the institute than by home-based care (see also Hoole 2012).

### Daily life and experiences at the institute

The exploration of the children’s life and experiences at the institute aims to focus on the casebook entries that provide an awareness and richer appreciation of the humanness and personhood of the children. This discussion draws on the works of Bogdan and Taylor (1989) and Eva Feder Kittay (1999, 2001, 2005a, 2005b, 2009, 2019) as invaluable resources for articulating and affirming the individuality and humanness of each child.

The institute’s casebook is replete with accounts of children who appealed for love and expressed delight at receiving affection. For example, Leigh Hewitt (HGM 24:59) would often come up to one, ‘wishing to be embraced and fondled’. By highlighting the children’s capacity for love, we develop an understanding of their humanness and personhood (see Kittay 1999, 2019). Some children actively solicited the attention and affection of adults by various means. The casebook describes that James Charman (HGM 24:73) ‘likes to be taken notice of’ and would become animated when visitors entered the institute, so as to gain their attention. Claudia Donaldson (HGM 24:78) aimed to attract attention from the staff and gain their affection by making ‘much fuss over small things’. For example, she would ‘hold out her hand to one and scream to attract attention to the cause of the weeping’, which would ‘perhaps be an almost imperceptible scratch’. Lastly, Celine Colley (HGM 24:87) would exhibit jealousy if the ‘nurse pays attention to the other children’.

The casebook contains evidence that the children were also capable of showing appreciation and affection to the institute’s staff. Suzanne Bezuidenhout (HGM 24:45) was described as affectionate, and Anri Barnard (HGM 24:91) was deemed to have ‘some power of affection’. During her 9 years at the institute, Suzanne Bezuidenhout (HGM 24:45) was ‘[*a*]ppreciative of everything done for her’, and Nico Viljoen (HGM 24:48) was described as appreciative of the kindnesses he received. This finding that the children showed affection and appreciation to the staff indicates that they were ‘reciprocating or giving back something important’ (Bogdan & Taylor 1989:144) in their relations with others.

Several of the children who were older and more capable would assist in caring for the children who were young, weak or less able (see also Hoole 2012:214). Kate Holgate (HGM 24:20) assisted by dressing some of the children, Mark Rawling (HGM 24:41) was an aid to ‘feeble children’, Edward Bainbridge (HGM 24:64) cared for the ‘helpless children’ and Tiaan Bezuidenhout (HGM 24:46) offered support by looking after a number of the children. Although these narratives are silent on the quality of care offered by the children, the casebook for Edward Bainbridge (HGM 24:64) describes that he was ‘kind to the younger children’ and that he ‘looks after them well’.

The institute provided a number of amusements and activities, such as a playground (G27–1896), picnics (G55–1904) and the sport and entertainment offered by the asylum (G28–1898; G60–1903). In addition to these, the institute’s casebook contains numerous entries about the children’s enjoyment of music and their attendance of the asylum’s weekly dances. For Bogdan and Taylor (1989:142), one dimension to recognise and comprehend the individuality of a person is to understand that they have ‘likes and dislikes’, ‘tastes and preferences’. In the institute’s casebook, we witness the distinct and individual musical and dance interests of the children. Jacques Swanepoel (HGM 24:26) ‘liked to hear the piano’, and Sabrina Stone (HGM 24:49) took an interest in singing. Ethan Johnson (HGM 24:79) attended the dances at the asylum, and when he returned to the institute, he would often spend his time trying to dance and sing.

By scrutinising the institute’s casebook, it is possible to argue that the children’s interest in music and dance had a bearing on the doctors’ concluding that they not only had a capacity for happiness, but were also capable of cherishing life and savouring its pleasures (see Kittay 1999:151–152). In contrast to the description of Rozelle Engelbrecht (HGM 24:80) as having ‘limited intelligence’, the doctors marvelled at her having a ‘wonderful idea of music’ and being able to ‘strum a tune on […] the piano’. Rozelle ‘picked up a lot of songs from the nurses’, but owing to her shyness, she would only sing them after much coaxing. In later entries, the doctors recorded that Rozelle had ‘increased her musical repertoire’ and that she ‘[*s*]eems happy’. As previously outlined, Ruan Odendaal (HGM 24:7) arrived at the institute in a ‘very dirty and neglected looking’ condition, having been found by the authorities to be locked up in his father’s house and left unattended. Whilst at the institute, he made no progress in his lessons, and his speech remained undeveloped, but he was regarded as a ‘happy little fellow, always singing’, and ‘he seems to have an excellent ear for music’. Greenlees conceded in the casebook that if one were to compare Ruan’s ‘relatively happy condition’ with how he used to be, it was evident that he ‘has improved having found pleasant surroundings’. Overall, Greenlees regarded Ruan as ‘happy enough in his own way’. Lastly, Tiaan Bezuidenhout’s (HGM 24:46) attendance and enjoyment of the weekly dances hosted by the asylum facilitated a doctor’s apprehension that he was ‘quite capable of enjoying life’.

For the children who were profoundly mentally and/or multiply disabled, they are presented in the institute’s casebook as objects of the doctors’ ‘dehumanising stigmatising gaze’ (Kittay 2005b:117). Nevertheless, the casebook also contains a number of entries that have the potential to highlight the personhood of the children.

In August 1898, before recommending that Christine Thompson (HGM 24:14) be transferred to the Chronic Sick Hospital, Greenlees presented a dehumanised description of her as ‘an interesting case pathologically but this is all. Educational abilities are *nil*; she is utterly unable to comprehend her lessons’ (emphasis in original). Yet, in the preceding entry, Greenlees did concede that Christine ‘smiles when spoken to’. In this regard, Eva Feder Kittay (2019:20) asserts that the ‘lack of expressive language’ in profoundly mentally and/or multiply disabled children ‘does not preclude the possibility of receptive language and understanding’. Along these lines, on scrutinising the casebook, it is evident that the children demonstrated an awareness of their environment and that they responded to personal engagement. Wessel le Roux (HGM 24:23) recognised and responded to the ‘sound of dishes’, whereas a number of children, such as Christine Thompson (HGM 24:14), were receptive and responsive to interpersonal encounters. For example, Danica Rose (HGM 24:27) could not speak but ‘laughs and grins […] when her attention is attracted’, and Shawn Murray (HGM 24:100), although he was unable to talk, would smile when looked at. Together, these findings demonstrate that the children were ‘far from being unresponsive to their environment and to other people’ (Kittay 2005a:126), and in foregrounding their ability to think and understand (Bogdan & Taylor 1989:139), their personhood is brought into view (Kittay 2001):

[*I*]n one who can scarcely move a muscle, a glint in the eye at a strain of familiar music establishes personhood. A slight upturn of the lip in a profoundly and multiply disabled individual when a favorite caregiver comes along, or a look of joy in response to the scent of a perfume – all these establish personhood. We know that there is a person before us when we see […] that there is ‘someone home’; that the seemingly vacuous look is not vacant at all; that an individual’s inability to articulate a ‘language’ as publicly defined does not indicate a lack of anything to say. (p. 568)

### Routes out of the institute

As the institute was established for CWID, in general this meant that once the children approached their late teens, they were transferred to other sites. In reviewing the institute’s casebook and annual reports, a more in-depth understanding emerges of the factors that informed the doctors’ decision to transfer the children. In particular, I will enumerate three factors that had a bearing on the length of time that a child stayed at the institute.

Firstly, for the children who were deemed by Greenlees to be ‘hopeless cases’ (G27–1896:39; G20–1897:24), he advocated that they be removed from the institute. For these children, Greenlees asserted that ‘there exists but little hope of any permanent mental or intellectual improvement in spite of all the care lavished upon them’ (G20–1897:25), and thus he sought quickly to transfer them to the Chronic Sick Hospital and the asylum, so that ‘accommodation will be provided to cases more amenable to our methods of education and treatment’ (G20–1897:25). On reviewing the casebook entries for these children, it is evident that the length of their stay at the institute was remarkably short, and their stay came to an end before they approached their late teens.

For her 3 years at the institute, Emma Rogers (HGM 24:8), a young teen who was blind and mute, has only one casebook entry, which outlines that ‘[*h*]er blindness prevents any progress in her education, and she sits quietly all day giving no trouble. Has to be fed and dressed’. Christiaan du Toit (HGM 24:57), aged 12, entered the institute in March 1904, with medical certificates stating that he was ‘almost blind’ and ‘deaf mute’. In the casebook he was dismissed as ‘utterly helpless so far as self help is concerned’, and he was transferred to the asylum in December 1907.

The second factor that informed the decision to transfer a child was displays of violent and troublesome behaviour. Put differently, once a child exhibited abusive, violent and uncontrollable behaviour, transfer from the institute was initiated. Although the institute made use of seclusion to manage troublesome behaviour (see HGM 24:48) and administered bromides to sedate unruly children (see HGM 24:73), the dominant approach used by the institute to deal with children who engaged in objectionable behaviour was to remove them by means of a transfer. For the children who spent a large portion of their childhood at the institute, the onset of violent behaviour in their teenage years marked the end of their time at the institute. Caitlin Page (HGM 24:17) was admitted in 1896 at the age of 7. In 1905, at the age of 16, when she began to show ‘violence to some of the children’, she was transferred to the asylum. The 10-year-old Gareth Dean (HGM 24:62) was admitted to the institute in September 1904. In 1910, he was transferred to the asylum on account of his impulsive behaviour, in which he would hit and bite the other children, and he even knocked over a nurse.

The third factor that influenced the length of a child’s stay at the institute was their ability to perform ‘useful work’ (HGM 24:48). For the children who provided labour, executed ward duties and cared for the other children, the duration of care that they received at the institute was long. It can thus be argued that the institute’s staff were biased towards children who were able to assist them, and this ensured that the children received preferential care and recreational offerings and meant that they were able to stay longer at the institute (see also Hoole 2012:240). By way of example, the siblings Suzanne (HGM 24:45) and Tiaan Bezuidenhout (HGM 24:46) were institutionalised for 9 years before being transferred to the Chronic Sick Hospital at the ages of 22 and 20, respectively. During their many years at the institute, they looked after the other children. Nico Viljoen (HGM 24:48) was 7 years old when he was admitted to the institute in April 1902. By 1913, when Nico was 18, the doctors conceded that he was ‘really too old for [*the institute*]’, but that he was ‘very useful there with the younger children’. It was only in May 1916, when Nico was 21, that he was transferred to Valkenberg Asylum.

In a number of cases, the children were discharged from the institute and were returned to the care of their families. For the purposes of this discussion, I will enumerate three dominant groups that constitute these cases. The first group is the children who made good progress in their education. To illustrate, Kate Holgate (HGM 24:20) took an interest in her lessons, and Greenlees explained that her ‘intellectual faculties have improved very much’, and that ‘her condition gives much satisfaction to her father’. After 6 years at the institute, Kate was discharged in 1902 to the care of her family. For several years thereafter, Kate’s family continued to report to the institute about her good health, her well-being and the sustained improvement that she was making in her lessons.

The second group is the children whose families were not satisfied with the progress their child was making at the institute. These families requested that their children be discharged from the institute so that they could seek alternative treatment options. Apart from the physical progress that Jacques Swanepoel (HGM 24:26) made by being able to walk, he improved very little at the institute. As his family were ‘not satisfied at his progress’, they were ‘anxious to remove him’ from the institute and to return to the hot springs that they had bathed Jacques in prior to his institutionalisation. Although Greenlees expressed ‘doubt as to the probability of improvement by bathing’, he informed the parents that ‘there is no reason why this shouldn’t be tried again’.

In January 1901, Kelsey Barker (HGM 24:42) was admitted to the institute, but owing to the unsatisfactory progress she made at the institute, her father requested in August 1902 that she be discharged. Greenlees lamented that he was informed that:

[*I*]t is the father’s intention having an operation performed on her head by a local medical man. This is being done without consultation with me and the father has been advised that it assuredly will not result in any mental benefit to the child and is likely to prove fatal (HGM 24:42).

It is not clear whether Kelsey was subjected to the surgery, but in October 1906 she was readmitted to the institute, and there are entries recorded for her until 1915 (see HGM 24:75).

The third group is the children who made no improvement at the institute, and their families elected to care for them at home. Hendrik Vermaak (HGM 24:43) made himself useful at the institute, but because he was deaf, he was unable to make progress in his lessons. After 3 years at the institute, Hendrik was discharged into his father’s care. Steven Hollamby (HGM 24:47) was described by Greenlees as a ‘disappointing case’, who has ‘not made the progress either mental or intellectual that was expected’. Steven was discharged to the care of his mother after only 1 year at the institute. Will Markham (HGM 24:54) was dismissed by Greenlees as being ‘quite incapable of learning anything’. Nevertheless, his family requested that he be discharged, and they were ‘prepared to give him a comfortable home and work to do’. During Rebecca Berman’s (HGM 24:88) short period of institutionalisation, which lasted only 7 months, she was disparaged by the doctors as being in a ‘wretched state’, yet this had no impact on the decision of her parents to be responsible for her care.

In these cases, the discharge of the children from the institute to the family reveals that their parents were committed to being responsible for their continued care and well-being (see also Clarke 2004/2005:65; Taylor 2017b:763).

With the opening of the Alexandra Hospital in 1921, the PWID who were still staying at the institute, and those who had been transferred to the asylum and Valkenberg Asylum, were all ultimately admitted to Alexandra Hospital, to become its first intake of patients (Foster 1990:36; Minde 1975b:1890).

## Conclusion

Contained in the casebook are glimpses of the life stories of the children before they were admitted to the institute. Although some of the children were sent to a number of sites before they arrived at the institute – including welfare establishments and hospitals – there is evidence that others experienced neglect and abuse by their families during their stay at home. Although these cases point to neglect, mistreatment and abandonment of the children by their families, this is only part of the story, and it in no way presents a dominant narrative of the children as ‘neglected waifs’ (G27–1896:39). The other part of the story highlights children who remained in the care of their families for a substantial part of their childhood, as well as families who sought a range of medical, surgical and alternative therapies prior to taking the decision to seek admittance for their child to the institute. By taking into consideration both parts of the story, we are presented with a heterogeneous picture of the children who were admitted to the institute. To this end, the article sought to identify the shared themes contained in the life stories of the children, as well as to draw attention to the unique aspects of an individual’s life story, so as to highlight the children’s individuality and humanness.

The casebook entries relating to the children’s time at the institute present evidence that they were responsive to the setting of the institute, and they were capable of interpersonal contact and relations (see also Kittay 2009). To illustrate, in the casebook the humanness of the children is portrayed, who demonstrated their ‘capacities for love and for happiness’ (Kittay 1999:152), by their appreciation of music, their care for other children and their expression of gratitude and affection. Based on this finding, it is possible to suggest that the children in their various routes out of the institute, to family care and to various other facilities, were able to live a life that was ‘richly human’ (Kittay 2005b:110).

By seeking to explore the life stories and the experiences of the CWID admitted to the Institute for Imbecile Children, this article reveals the life stories of only the ‘minority who resided within asylums’, rather than the life stories of ‘the majority who remained outside institutions’ (Clarke 2006:471). Although the article offers only a glimpse into the lives of South African CWID living in the late 19th and early 20th centuries, the findings offer a means to redress the existing negative narratives of the children at the institute, and to offer the children an identity ‘expressed in positive terms’ (Kittay 2019:6), by highlighting their capacity to live the ‘apotheosis of a good life’ (Kittay 2019:54), encapsulated by Kittay (2019:54) as being able ‘[*t*]o love, to derive joy from life, to learn the wonder of being’.

## Footnotes

1 *The Fort England Mirror* was published by Greenlees as one means to produce and disseminate a positive public image of the asylum and the institute, with the aim of increasing custom from paying patients, and to motivate the public to acts of charity and philanthropy for the institutionalised patients of the asylum, the institute, and the Chronic Sick Hospital. The contradiction between the positive public image of the institute, and Greenlees’ scientific publications, where he advocated for the extermination of PWID, is explored further in (Du Plessis 2021).

2 See also Klausen (1997).

3 More broadly, Greenlees’ belief in eugenics is evident in him proclaiming that the ‘two great factors in the causation of mental disease are: a hereditary predisposition to mental instability; and intemperance in the use of alcoholic stimulants’ (Greenlees 1905:223). The solution to put an end to insanity lay for Greenlees in preaching teetotalism and ‘prohibiting injudicious marriages’ (Greenlees 1903:19) between couples who had a family history of mental illness. In terms of the latter point, Greenlees called for the prevention of insanity by urging the government to ‘prohibit by legal enactment the marriage of cousins – one of the most prolific causes of imbecility’ (Greenlees 1907:17), and he called on general practitioners to condemn marriage between people who were suffering from hereditary physical and/or mental illness.

4 The Chronic Sick Hospital was established in 1882 and admitted impoverished pensioners, as well as infirm, destitute and starving individuals (Du Plessis 2017).
