# Erratum: The life stories and experiences of the children admitted to the Institute for Imbecile Children from 1895 to 1913

**DOI:** 10.4102/ajod.v10i0.812

**Published:** 2021-08-02

**Authors:** Rory du Plessis

**Affiliations:** 1 School of the Arts, Faculty of Humanities, University of Pretoria, Pretoria, South Africa

In the version of this article initially published, Du Plessis, R., 2020, ‘The life stories and experiences of the children admitted to the Institute for Imbecile Children from 1895 to 1913’, *African Journal of Disability* 9(0), a669. https://doi.org/10.4102/ajod.v9i0.669, the author’s affiliation was given incorrectly in the ‘Affiliation’ section. The correct affiliation should be ‘School of the Arts, Faculty of Humanities, University of Pretoria, Pretoria, South Africa’ instead of ‘School of Visual Arts, Faculty of Humanities, University of Pretoria, Pretoria, South Africa’.

This correction does not alter the significance of the study findings or the overall interpretation of the study results. The publisher apologises for any inconvenience caused.

